# Association of chemotherapy with survival in stage II colon cancer patients who received radical surgery: a retrospective cohort study

**DOI:** 10.1186/s12885-021-08057-3

**Published:** 2021-03-23

**Authors:** Zhihao Lv, Yuqi Liang, Huaxi Liu, Delong Mo

**Affiliations:** 1 Proctology Department, Zhongshan Hospital Affiliated to Guangzhou University of Chinese Medicine, No. 3 Kangxin Road, West District, Zhongshan, 528400 Guangdong People’s Republic of China; 2 Science and Technology Innovation Center, Guangzhou University of Chinese Medicine, Guangzhou, Guangdong People’s Republic of China; 3 College of Traditional Chinese Medicine, Southern Medical University, Guangzhou, Guangdong People’s Republic of China

**Keywords:** Colon cancer, SEER, Chemotherapy, Nomogram, Competing-risk model

## Abstract

**Background:**

It remains controversial whether patients with Stage II colon cancer would benefit from chemotherapy after radical surgery. This study aims to assess the real effectiveness of chemotherapy in patients with stage II colon cancer undergoing radical surgery and to construct survival prediction models to predict the survival benefits of chemotherapy.

**Methods:**

Data for stage II colon cancer patients with radical surgery were retrieved from the Surveillance, Epidemiology, and End Results (SEER) database. Propensity score matching (1:1) was performed according to receive or not receive chemotherapy. Competitive risk regression models were used to assess colon cancer cause-specific death (CSD) and non-colon cancer cause-specific death (NCSD). Survival prediction nomograms were constructed to predict overall survival (OS) and colon cancer cause-specific survival (CSS). The predictive abilities of the constructed models were evaluated by the concordance indexes (C-indexes) and calibration curves.

**Results:**

A total of 25,110 patients were identified, 21.7% received chemotherapy, and 78.3% were without chemotherapy. A total of 10,916 patients were extracted after propensity score matching. The estimated 3-year overall survival rates of chemotherapy were 0.7% higher than non- chemotherapy. The estimated 5-year and 10-year overall survival rates of non-chemotherapy were 1.3 and 2.1% higher than chemotherapy, respectively. Survival prediction models showed good discrimination (the C-indexes between 0.582 and 0.757) and excellent calibration.

**Conclusions:**

Chemotherapy improves the short-term (43 months) survival benefit of stage II colon cancer patients who received radical surgery. Survival prediction models can be used to predict OS and CSS of patients receiving chemotherapy as well as OS and CSS of patients not receiving chemotherapy and to make individualized treatment recommendations for stage II colon cancer patients who received radical surgery.

## Background

Colon cancer is one of the most common malignant diseases, and it is estimated that 53,200 Americans will die from colon cancer in 2020, equivalent to more than 145 deaths per day [1]. Approximately 30–40% of colon cancer patients belong to stage II and they are recommended for radical surgery [2–4]. Although chemotherapy is widely accepted as the standard treatment for stage III colon cancer to prevent recurrence and metastasis, controversy remains over whether stage II colon cancer should receive it after radical surgery.

Both The American Society of Clinical Oncology (ASCO) clinical guidelines [5] and The European Society for Medical Oncology (ESMO) guidelines [6] recommend patients with high-risk factors to receive postoperative adjuvant chemotherapy. These high-risk factors include inadequate number of resected lymph nodes, T4 primary tumor, histology of poorly differentiated, bowel obstruction or perforation, and lymphovascular invasion. Two recent studies [7, 8] based on more than 100,000 patients, both concluded that all patients with stage II colon cancer, whether high or low risk, experienced significant improvements in overall survival (OS) associated with receiving chemotherapy, while some clinical studies showed that chemotherapy failed to improved survival [9, 10].

Previous studies had explored the importance of covariates in identifying prognostic, but most of them failed to clearly state whether patients underwent surgery and which type of surgery they received.

Consequently, this study aimed to construct nomograms as survival prediction models and to individualized assess the potential survival benefit of stage II colon cancer patients with chemotherapy after radical surgery.

## Methods

### Data source

This was a retrospective study. Data from patients with stage II colon cancer (one primary only) between 2004 and 2015 was extracted from a total of 18 cancer registries utilizing the National Cancer Institute’s SEER Cancer database (1973–2016) using the SEER*Stat software version 8.3.6. We received permission to access the research data (Account Number: 20532-Nov2018) and chemotherapy information was obtained by submitting a special data request to the SEER program. This study was based on public data and permission was obtained to access this data for research only. This was not an interventional study and did not use personal identifying information. Therefore, informed consent for the study was not required. The authors have no conflicts of interest to declare. This study has been registered as a retrospective study at the Chinese Clinical Trial Registry (ChiCTR) with registration number as ChiCTR2000031512. All procedures performed in our study were in line with the STROCSS criteria [11].

### Patient selection

**Fig. 1:**
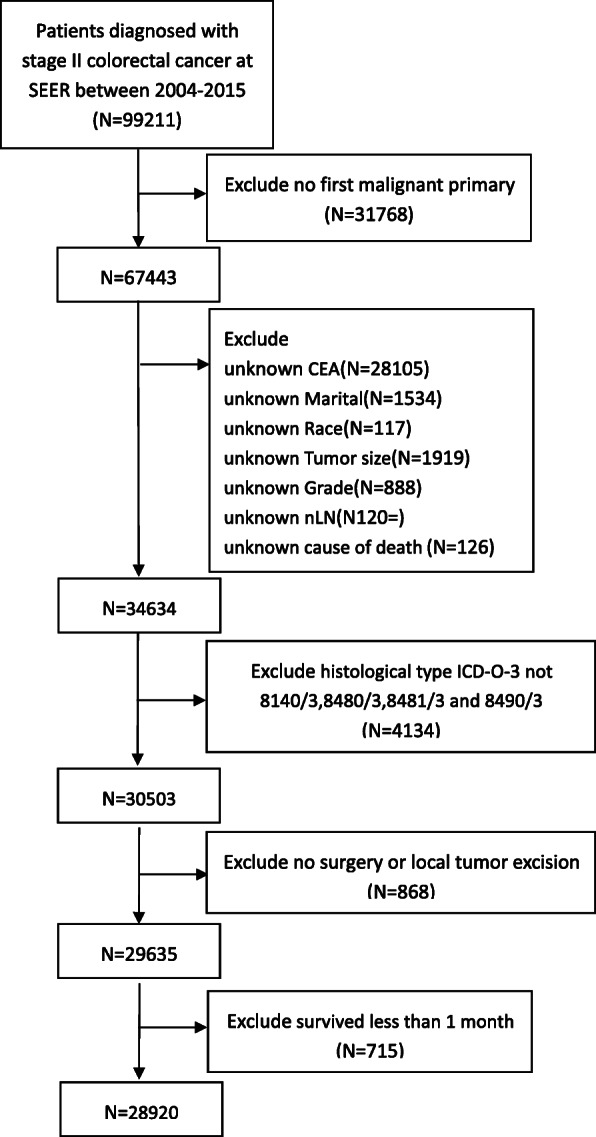
Flow chart of the patient selection process

The exclusion criteria were as follows: a) the clinicopathological information was incomplete; b) more than one primary tumor; c) unknown cause of death; d) histological type ICD-O-3, not 8140/3, 8480/3, 8481/3 and 8490/3; e) no surgery or local tumor excision only; f) died within 30 days of surgery. Finally, 25,110 patients were included for analysis (Fig. 1).

Individual data retrieved for each case included age at diagnosis, sex, marital status, race, tumor site, tumor size, histology, grade, T stage, Number of lymph nodes (nLN), carcinoembryonic antigen (CEA), year of diagnosis, surgery classification, chemotherapy recode, cause-specific death classification, vital status and survival months.

Marital status was regrouped as married or unmarried (single, widowed, divorced and separated). The tumor site was classified as the right colon (including the cecum, the ascending colon, the hepatic flexure and the transverse colon) and the left colon (including the splenic flexure and the descending, sigmoid colons and rectosigmoid junction). Number of lymph nodes (nLN) sampled was regrouped as 0, 1–11, ≥12 and tumor size was regrouped as < 5 cm, ≥5 cm according to the X-tile program [12]. The variable chemotherapy was classified as chemotherapy ‘yes’ or ‘no/unknown’ by the SEER program [13].

### Outcomes

The outcomes of this study were overall survival (OS) and colon cancer cause-specific survival (CSS). OS was defined as the time from diagnosis to the date of death due to any cause. CSS was defined as the time from diagnosis to the date of death due to colon cancer. Colon cancer cause-specific death (CSD) and non-colon cancer cause-specific death (NCSD) were used in competing risk analysis. CSD was defined the same as CSS. NCSD was calculated from the time of diagnosis to the time of death due to causes other than colon cancer. Death attributed to colon cancer was regarded as the failure event. Patients who died from other causes was the competing event, and vice versa.

### Statistical analysis

To mimic the randomized controlled trials and balance important patient characteristics between groups, a 1-to-1 propensity score matching (PSM) method was performed with the nearest-neighbor method. Differences in distributions of chemotherapy subgroups were assessed with Chi-squared tests. Cumulative incidence was calculated by a competing risk model and a cumulative incidence plot was constructed to describe the actual prognosis of different causes of death. Univariate and multivariate cox proportional hazard models with hazard ratio (HR) and 95% confidence interval (CI) for OS and CSS were performed. Clinicopathological variables with *p* values < 0.05 in the univariate analysis were selected into the multivariate analysis. The variable chemotherapy was also selected based on clinical significance, scientific knowledge and predictors identified in previously published articles [14–17]. Nomograms were constructed in R (version 3.6.2) and subjected to 1000 bootstrap resamples for internal validation. The concordance indexes (C-indexes) were calculated and calibration plots were performed to assess the predictive accuracy of OS and CSS. All statistical analyses were performed using SPSS version 25 (IBM Corporation, Armonk, NY, USA) and *p* values < 0.05 were considered statistically significant.

## Results

### Clinical characteristics of patients and survival outcomes

A total of 25,110 patients with stage II colon cancer were included from the SEER database. The median follow-up time was 80 months (range 1–155 months). Before propensity score matching, 5458 (21.7%) patients received chemotherapy, and 19,652 (78.3%) patients were without chemotherapy. After propensity score matching, patients with chemotherapy were older, more often female, more often unmarried, more often White, had more often left colon cancer, had bigger tumors, presented more often with advanced T classification, had fewer lymph nodes and presented more often with positive CEA. The baseline characteristics of patients stratified by chemotherapy are listed in Table 1.

**Table 1:** Baseline characteristics of chemotherapy subgroups before and after propensity score matching

	non-chemotherapy	Chemotherapy		non-chemotherapy	Chemotherapy	
Variable	***n*** = 19,652 (%)	***n*** = 5458 (%)	***p***	n = 5458 (%) (PSM)	n = 5458(%) (PSM)	***p***
Age			0.000			0.000
> 30	57 (0.3)	57 (1.0)		57 (1.0)	57 (1.0)	
30–39	227 (1.2)	284 (5.2)		226 (4.1)	284 (5.2)	
40–49	1033 (5.3)	879 (16.1)		918 (16.8)	879 (16.1)	
50–59	2831 (14.4)	1574 (28.8)		1640 (30.0)	1574 (28.8)	
60–69	4380 (22.3)	1552 (28.4)		1407 (25.8)	1552 (28.4)	
70–79	5355 (27.2)	906 (16.6)		871 (16.0)	906 (16.6)	
≥ 80	5769 (29.4)	206 (3.8)		339 (6.2)	206 (3.8)	
Sex			0.000			0.730
Female	10,282 (52.3)	2576 (47.2)		2558 (46.9)	2576 (47.2)	
Male	9370 (47.7)	2882 (52.8)		2900 (53.1)	2882 (52.8)	
Marital status			0.000			0.036
Married	10,325 (52.5)	3361 (61.6)		3467 (63.5)	3361 (61.6)	
Unmarried^a^	9327 (47.5)	2097 (38.4)		1991 (36.5)	2097 (38.4)	
Race			0.098			0.924
White	15,736 (80.1)	4314 (79.0)		4299 (78.8)	4314 (79.0)	
Black	2114 (10.8)	643 (11.8)		647 (11.9)	643 (11.8)	
Other	1802 (9.2)	501 (9.2)		512 (9.4)	501 (9.2)	
Tumor site			0.000			0.700
Right colon^b^	12,128 (61.7)	2423 (44.4)		2443 (44.8)	2423 (44.4)	
Left colon^c^	7524 (38.3)	3035 (55.6)		3015 (55.2)	3035 (55.6)	
Tumor size			0.000			0.007
<5	9649 (49.1)	2302 (42.2)		2441 (44.7)	2302 (42.2)	
≥ 5	10,003 (50.9)	3156 (57.8)		3017 (55.3)	3156 (57.8)	
Histology			0.136			0.254
Adenocarcinoma	17,172 (87.4)	4768 (87.4)		4816 (88.2)	4768 (87.4)	
Mucinous adenocarcinoma	2374 (12.1)	648 (11.9)		610 (11.2)	648 (11.9)	
Signet ring cell carcinoma	106 (0.5)	42 (0.8)		32 (0.6)	42 (0.8)	
Grade			0.000			0.005
I	1311 (6.7)	341 (6.2)		282 (5.2)	341 (6.2)	
II	15,192 (77.3)	4050 (74.2)		4196 (76.9)	4050 (74.2)	
III	2818 (14.3)	943 (17.3)		853 (15.6)	943 (17.3)	
IV	331 (1.7)	124 (2.3)		127 (2.3)	124 (2.3)	
T stage			0.000			0.000
T3	17,663 (89.9)	3919 (71.8)		4279 (78.4)	3919 (71.8)	
T4	1989 (10.1)	1539 (28.2)		1179 (21.6)	1539 (28.2)	
nLN^d^			0.000			0.064
0	92 (0.5)	56 (1.0)		35 (0.6)	56 (1.0)	
1–11	3507 (17.8)	1056 (19.3)		1093 (20.0)	1056 (19.3)	
≥ 12	16,053 (81.7)	4346 (79.6)		4330 (79.3)	4346 (79.6)	
CEA^e^			0.000			0.026
Positive	7367 (37.5)	2212 (40.5)		2098 (38.4)	2212 (40.5)	
Negative	12,285 (62.5)	3246 (59.5)		3360 (61.6)	3246 (59.5)	

### Cumulative incidence of death and competing risk analysis

**Fig. 2:**
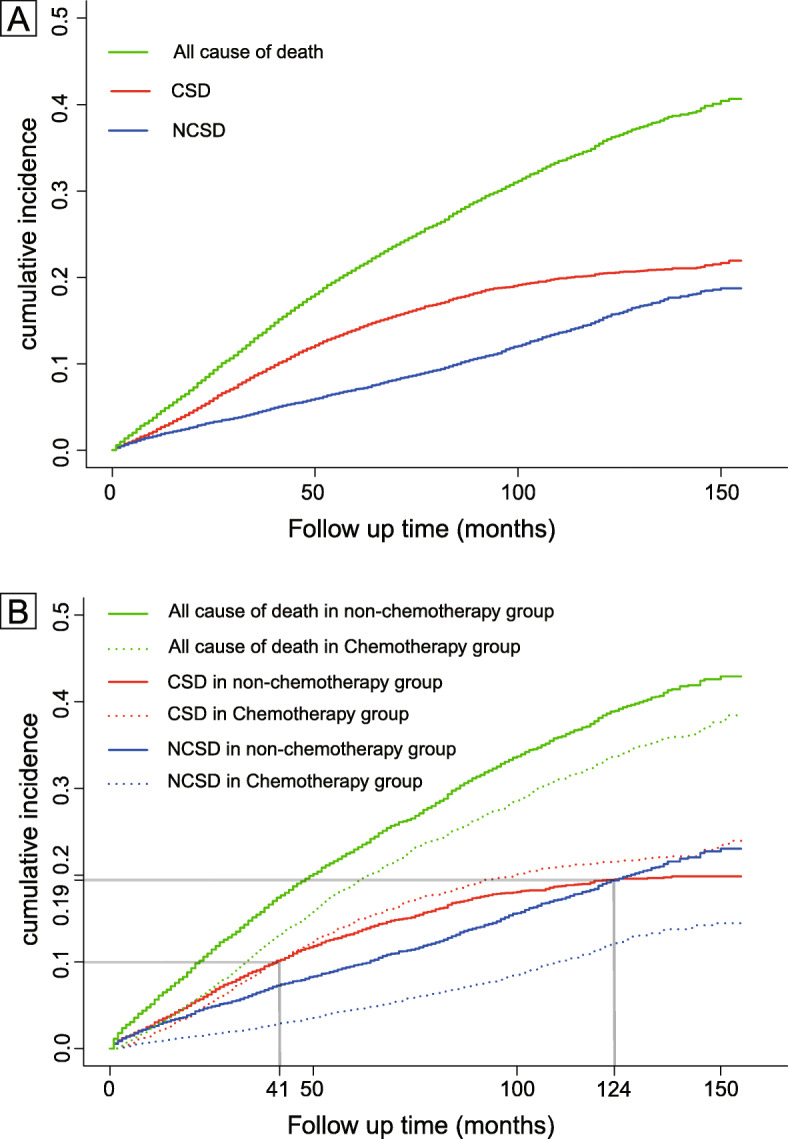
Competing risk models. **a**: Stacked cumulative incidence plots. **b**: CSD, NCSD and all cause of death of receiving or not receiving chemotherapy subgroups. Patients with chemotherapy showed higher all cause of death, CSD, and NCSD than patients who did not receive chemotherapy after 43, 40, and 52 months follow up, respectively. Abbreviations: CSD, colon cancer cause-specific death; NCSD, non-colon cancer cause-specific death

A total of 2334 (21.3%) patients died, of which 1628 (69.7%) died from colon cancer and 706 (30.2%) died from causes other than colon cancer. The estimated 3-year overall survival rates of chemotherapy were 0.7% higher than non-chemotherapy. The estimated 5-year and 10-year overall survival rates of non-chemotherapy were 1.3 and 2.1% higher than chemotherapy, respectively. The 3-, 5-, and 10-year cumulative incidence of CSD, NCSD and all cause of death are shown in Table 2. Patients with chemotherapy showed higher all cause of death, CSD, and NCSD than patients who did not receive chemotherapy after 43, 40, and 52 months follow up, respectively (Fig. 2).

**Table 2:** The 3-, 5-, and 10-year cumulative incidence of CSD, NCSD and all cause of death

	All patients(%)	Chemotherapy	
		Yes(%)	No(%)
All cause of death			
3-Year CIF^a^	11.2	10.8	11.5
5-Year CIF	17.3	17.9	16.6
10-Year CIF	29.5	30.5	28.4
CSD^b^			
3-Year CIF	8.8	8.7	8.9
5-Year CIF	13.4	14.0	12.7
10-Year CIF	19.8	20.1	19.4
NCSD^c^			
3-Year CIF	2.4	2.1	2.6
5-Year CIF	3.9	3.9	3.9
10-Year CIF	9.8	10.5	9.1

^a^*CIF* cumulative incidence function; ^b^*CSD* colon cancer cause-specific death; ^c^*NCSD* non-colon cancer cause-specific death

### Univariate and multivariate analyses of OS and CSS

Univariate and multivariate cox regressions were performed to identify the variables associated with OS and CSS. In univariate survival analysis, sex showed significant association with OS, but no statistical relationship with CSS. Other variables including age, marital status, race, tumor site, tumor size, histology, grade, T stage, nLN and CEA were proved to be significantly correlated with OS and CSS (*p* < 0.05). All variables that were classified as statistically significant in univariate analysis were included in multivariate analysis and used to perform the nomogram to predict 3-, 5- and 10-year OS and CSS rates (Table 3).

**Table 3:** OS and CSS in univariate and multivariate analyses

Risk factors	OS				CSS			
	Univariate analyses		Multivariate analyses		Univariate analyses		Multivariate analyses	
	HR (95% CI)	***p***	HR (95% CI)	***p***	HR (95% CI)	***p***	HR (95% CI)	***p***
Age		0.000				0.000		
> 30	1	–	1	–	1	–	1	–
30–39	1.71 (0.77–3.77)	–	1.75 (0.79–3.87)	0.166	1.39 (0.63–3.11)	–	1.37 (0.61–3.05)	0.447
40–49	1.86 (0.88–3.96)	–	1.90 (0.89–4.05)	0.095	1.48 (0.69–3.15)	–	1.46 (0.68–3.11)	0.332
50–59	2.55 (1.21–5.38)	–	2.44 (1.15–5.14)	0.020	1.94 (0.92–4.10)	–	1.76 (0.83–3.72)	0.139
60–69	3.80 (1.80–7.99)	–	3.47 (1.65–7.32)	0.001	2.63 (1.24–5.54)	–	2.25 (1.06–4.75)	0.034
70–79	6.63 (3.15–13.96)	–	5.57 (2.64–11.74)	0.000	4.07 (1.93–8.60)	–	3.11 (1.47–6.57)	0.003
≥ 80	12.97 (6.13–27.44)	–	9.96 (4.70–21.10)	0.000	7.58 (3.57–16.12)	–	5.14 (2.41–10.95)	0.000
Sex		0.024				0.902		
Female	1	–	1	–	1	–	–	–
Male	1.10 (1.01–1.19)	–	1.32 (1.21–1.44)	0.000	1.01 (0.91–1.11)	–	–	–
Marital status		0.000				0.000		
Married	1	–	1	–	1	–	1	–
Unmarried^a^	1.57 (1.45–1.71)	–	1.33 (1.22–1.45)	0.000	1.58 (1.43–1.74)	–	1.27 (1.14–1.40)	0.000
Race		0.000				0.000		
White	1	–	1	–	1	–	1	–
Black	1.29 (1.15–1.45)	–	1.41 (1.26–1.59)	0.000	1.41 (1.23–1.61)	–	1.47 (1.28–1.69)	0.000
Other	0.73 (0.62–0.86)	–	0.77 (0.65–0.90)	0.001	0.86 (0.71–1.03)	–	0.88 (0.73–1.05)	0.161
Tumor site		0.048				0.000		
Right colon^b^	1	–	1	–	1	–	1	–
Left colon^c^	1.09 (1.00–1.18)	–	1.14 (1.04–1.24)	0.003	1.21 (1.09–1.33)	–	1.25 (1.13–1.39)	0.000
Tumor size		0.001				0.016		
<5	1	–	1	–	1	–	1	–
≥ 5	1.15 (1.06–1.25)	–	1.03 (0.95–1.12)	0.494	1.13 (1.02–1.25)	–	0.99 (0.89–1.10)	0.864
Histology		0.000				0.001		
Adenocarcinoma	1	–	1	–	1	–	1	–
Mucinous adenocarcinoma	1.14 (1.01–1.28)	–	0.97 (0.86–1.10)	0.668	1.14 (0.99–1.32)	–	1.00 (0.87–1.16)	0.964
Signet ring cell carcinoma	2.09 (1.43–3.06)	–	1.34 (0.91–1.98)	0.138	2.20 (1.42–3.42)	–	1.45 (0.92–2.28)	0.105
Grade		0.000				0.020		
I	1	–	1	–	1	–	1	–
II	1.00 (0.84–1.19)	–	1.06 (0.88–1.26)	0.553	0.98 (0.79–1.20)	–	1.04 (0.84–1.28)	0.750
III	1.19 (0.98–1.45)	–	1.13 (0.93–1.38)	0.226	1.15 (0.92–1.45)	–	1.13 (0.90–1.44)	0.299
IV	1.54 (1.14–2.08)	–	1.54 (1.14–2.09)	0.005	1.33 (0.92–1.92)	–	1.37 (0.95–1.99)	0.095
T stage		0.000				0.000		
T3	1	–	1	–	1	–	1	–
T4	2.88 (2.65–3.13)	–	2.21 (2.02–2.41)	0.000	3.20 (2.90–3.53)	–	2.59 (2.33–2.87)	0.000
nLN^d^		0.000				0.000		
0	1	–	1	–	1	–	1	–
1–11	0.52 (0.39–0.70)	–	0.56 (0.41–0.75)	0.000	0.41 (0.30–0.57)	–	0.48 (0.34–0.66)	0.000
≥ 12	0.31 (0.23–0.42)	–	0.38 (0.28–0.51)	0.000	0.25 (0.19–0.35)	–	0.33 (0.24–0.45)	0.000
CEA^e^		0.000				0.000		
Positive	1	–	1	–	1	–	1	–
Negative	0.51 (0.47–0.55)	–	0.62 (0.57–0.67)	0.000	0.50 (0.45–0.55)	–	0.62 (0.56–0.69)	0.000

^a^Unmarried, including single, widowed, divorced and separated;

^b^Right colon, including the cecum, the ascending colon, the hepatic flexure and the transverse colon;

^c^Left colon, including the splenic flexure and the descending, sigmoid colons and rectosigmoid junction;

^d^*nLN* number of lymph nodes; ^e^*CEA* carcinoembryonic antigen

### Nomogram

**Fig. 3:**
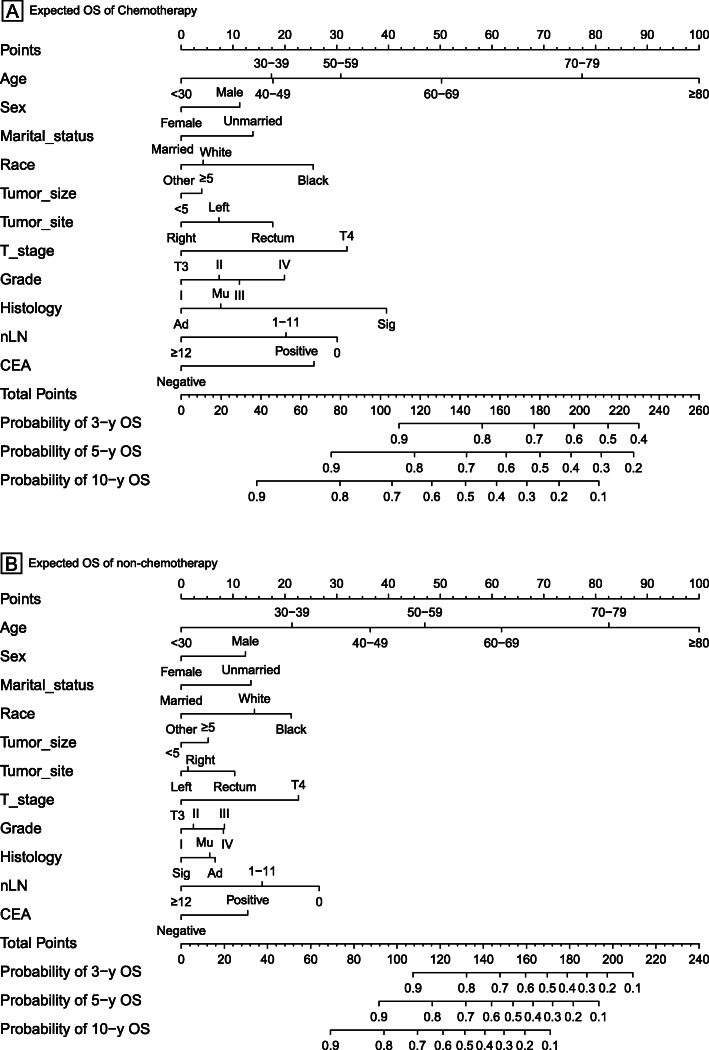
Nomograms for comparing expected 3-, 5-, and 10-year overall survival (OS) with and without chemotherapy of stage II colon cancer patients received radical surgery. Abbreviations: Ad, Adenocarcinoma; Mu, Mucinous adenocarcinoma; Sig, Signet ring cell carcinoma; Right colon, including the cecum, the ascending colon, the hepatic flexure and the transverse colon; Left colon, including the splenic flexure and the descending, sigmoid colons and rectosigmoid junction; nLN, number of lymph nodes; CEA, carcinoembryonic antigen

**Fig. 4:**
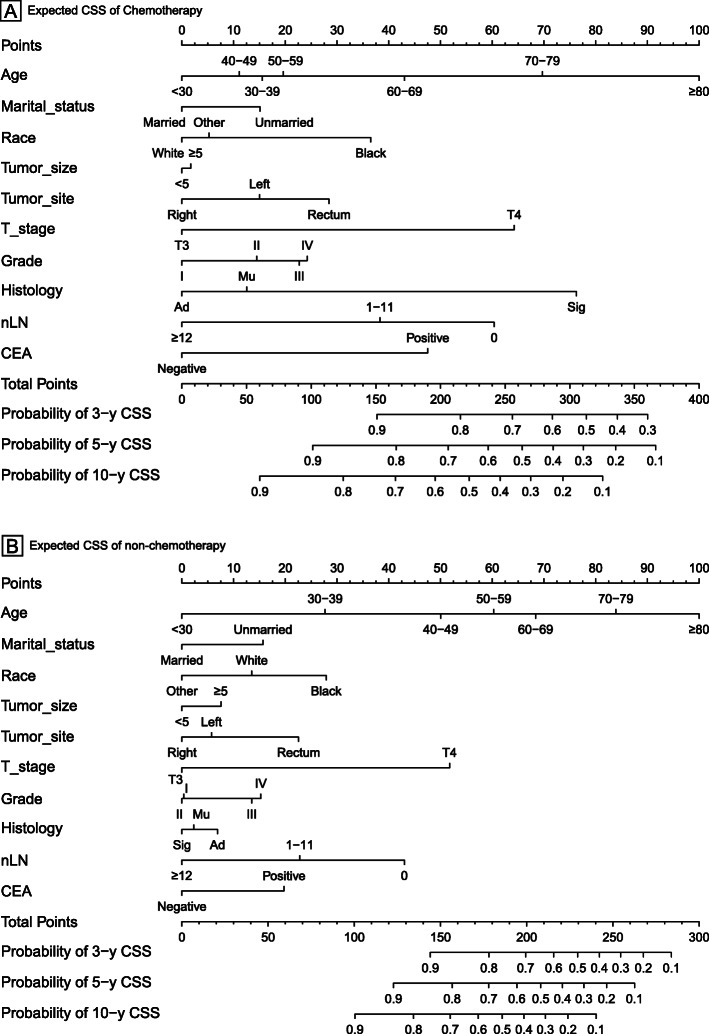
Nomograms for comparing expected 3-, 5-, and 10-year colon cancer cause-specific survival (CSS) with and without chemotherapy of stage II colon cancer patients received radical surgery. Abbreviations: Ad, Adenocarcinoma; Mu, Mucinous adenocarcinoma; Sig, Signet ring cell carcinoma; Right colon, including the cecum, the ascending colon, the hepatic flexure and the transverse colon; Left colon, including the splenic flexure and the descending, sigmoid colons and rectosigmoid junction; nLN, number of lymph nodes; CEA, carcinoembryonic antigen

**Fig. 5:**
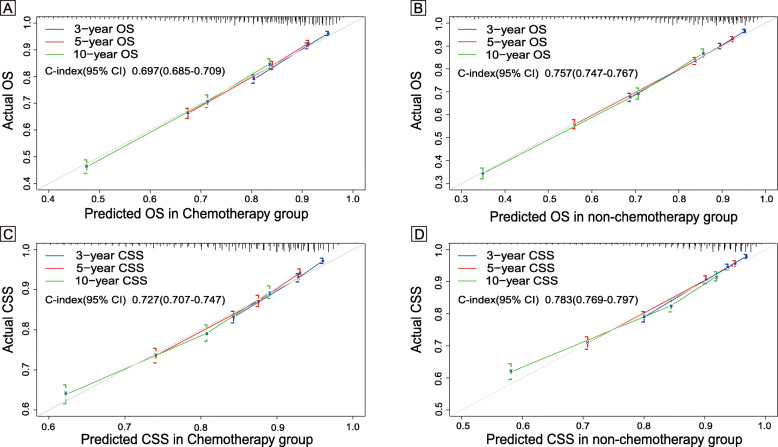
Calibration curve of the nomograms. **a**: 3-, 5-, and 10-year OS nomogram calibration curves of the chemotherapy group. **b**: 3-, 5-, and 10-year OS nomogram calibration curves of the non-chemotherapy group. **c**: 3-, 5-, and 10-year CSS nomogram calibration curves of the chemotherapy group. **d**: 3-, 5-, and 10-year CSS nomogram calibration curves of the non-chemotherapy group. Abbreviations: OS, overall survival; CSS, colon cancer cancer-specific survival

The nomograms for chemotherapy and non-chemotherapy were built to predict 3-, 5- and 10-year OS and CSS (Figs. 3, 4). The C-indexes for chemotherapy and non-chemotherapy to predict OS were 0.711 and 0.757, respectively (95% CI, 0.697–0.725 and 0.743–0.771) and the C-indexes for chemotherapy and non-chemotherapy to predict CSS were 0.582 and 0.589, respectively (95% CI, 0.572–0.592 and 0.579–0.599). The calibration plots showed an excellent correlation between the nomogram predicted and the outcome observed (Fig. 5).

## Discussion

This study constructed survival prediction nomograms that could effectively predict the OS and CSS of chemotherapy or non-chemotherapy after radical surgery for stage II colon cancer patients. The model accurately identifies patients who can benefit from chemotherapy and assists in making individual recommendations. The model is an extension of the stage grouping in the AJCC Cancer Staging Manual and can be effectively applied in clinical practice with good discrimination (the C-indexes between 0.582 and 0.757) and excellent calibration. Although some nomograms have been developed to predict the individual survival probability of colon cancer patients, there are still some unique features in our model [14–17]. Firstly, patients with stage II colon cancer were accurately included as the study participants based on particular chemotherapy controversy. Secondly, only patients receiving radical surgery could be included in the study to ensure homogenization. Thirdly, we included as many prognostic factors as possible based on clinical significance and statistical methods, and X-tile were used to determine the grouping of variables. Finally, in addition to overall survival, colon cancer cause-specific survival was also reported to predict the individual survival probability of patients to avoid the influence of additional unmeasured confounder related to the patient’s state of health.

However, although the potential survival benefit predicted by the model is an important consideration, it should not be the sole basis for decision making. Quality of life, economic conditions, and specific preferences of patients are also important factors in making treatment decisions.

Controversy exists regarding whether patients with stage II colon cancer after radical surgery should receive chemotherapy. Previous research findings were divided into three categories: a) recent studies showed chemotherapy provided a survival benefit to all stage II colon cancer patients [7, 8]; b) another two studies reported chemotherapy did not substantially improve OS [18, 19]; c) the QUASAR trial [20] and a pooled analysis [21] reported the survival benefits of chemotherapy only in high-risk stage II colon cancer patients, and it had confirmed by JSCCR [22], ASCO [5], NCCN [23] and ESMO [24] guidelines. This study suggests that chemotherapy improves the short-term (43 months) survival benefit of stage II colon cancer patients who received radical surgery. However, this conclusion should be treated with caution. Firstly, this study included patients since 2004 to obtain long-term follow-up data (using the TNM classification in the sixth edition of the AJCC Cancer Staging Manual), but the definition of N1c was proposed in 2010 (the TNM classification in the seventh edition of the AJCC Cancer Staging Manual). Therefore, stage II patients we included inevitably contained some stage III patients (TXN1cM0). The conclusion that patients with stage III colon cancer benefit from chemotherapy is now widely accepted [25, 26]. Consequently, patients included in this study are more likely to be registered as positive chemotherapy results if they are mixed with unidentified stage III patients. Secondly, OS is widely accepted as the primary outcome measure in clinical studies. OS is based on the absolute risk of death without considering the specific cause of death of patients, and patients with stage II colon cancer have a long-term life expectancy. Thus, they are inevitably at high risk of NCSD. Our results show that although the risk of CSD is always higher than the risk of NCSD, both the risks are very close when the patient’s expected survival time reaches 10 years or more. Therefore, the improvement in the overall survival of the chemotherapy group cannot simply be interpreted as the benefit of chemotherapy. Why, then, do patients receiving chemotherapy always have a higher risk of dying from colon cancer than from other causes. On the one hand, patients receiving chemotherapy have a better physical condition, signifying a lower risk of NCSD. On the other hand, the toxicity caused by chemotherapy improves the risk of CSD.

This study has several limitations. Firstly, some molecular biomarkers such as microsatellite instability (MSI) and BRAF mutations that could affect the prognosis of stage II colon cancer were not included in our analysis, which might lead to a certain degree of bias [27]. Secondly, the chemotherapy information in SEER database inevitably causes a confounding bias (72.1% sensitivity according to studies) [13]; for example, the chemotherapy record in SEER database is classified as “No/Unknown” and “Yes”. Although we obtained data of 5458 patients with definite chemotherapy from SEER database, we don’t know whether the patients recorded as “No/Unknown” actually received chemotherapy. Thirdly, we simply compared the chemotherapy group with the non-chemotherapy group and did not distinguish different chemotherapy regimens. Finally, this is a retrospective study that may introduce inherent selection bias.

## Conclusions

We develop a survival prediction model to estimate individual net survival benefit of chemotherapy in patients with stage II colon cancer after radical surgery. To the best of our knowledge, these nomograms are the first survival prediction models to predict the survival benefit of chemotherapy in stage II colon cancer patients after radical surgery using both OS and CSS outcome measures. This model can help clinicians to quantify the benefits of chemotherapy after radical surgery of stage II colon cancer patients and to make personalized treatment recommendations and decisions.

## Data Availability

The data used in this study are available free of charge online at www.seer.cancer.gov on request.

## Abbreviations

SEER: Surveillance, Epidemiology, and End Results
PSM: Propensity score matching
CSD: Colon cancer cause-specific death
NCSD: Non-colon cancer cause-specific death
OS: Overall survival
CSS: Colon cancer cause-specific survival
C-indexes: Concordance indexes
HR: Hazard ratio
CI: Confidence interval
